# Reversible Lineage-Specific Priming of Human Embryonic Stem Cells Can Be Exploited to Optimize the Yield of Differentiated Cells

**DOI:** 10.1002/stem.1952

**Published:** 2015-01-13

**Authors:** Jung Bok Lee, Monica Graham, Tony J Collins, Jong-Hee Lee, Seok-Ho Hong, Amie Jamie Mcnicol, Zoya Shapovalova, Mickie Bhatia

**Affiliations:** a McMaster Stem Cell and Cancer Research Institute, Michael G. DeGroote School of Medicine, McMaster UniversityHamilton, Ontario, Canada; b Department of Biochemistry, McMaster UniversityHamilton, Ontario, Canada

**Keywords:** Differentiation, Pluripotent stem cells, Self-renewal, Embryonic stem cells, Developmental biology

## Abstract

The clinical use of human embryonic stem cells (hESCs) requires efficient cellular expansion that must be paired with an ability to generate specialized progeny through differentiation. Self-renewal and differentiation are deemed inherent hallmarks of hESCs and a growing body of evidence suggests that initial culture conditions dictate these two aspects of hESC behavior. Here, we reveal that defined culture conditions using commercial mTeSR1 media augment the expansion of hESCs and enhance their capacity for neural differentiation at the expense of hematopoietic lineage competency without affecting pluripotency. This culture-induced modification was shown to be reversible, as culture in mouse embryonic fibroblast-conditioned media (MEF-CM) in subsequent passages allowed mTeSR1-expanded hESCs to re-establish hematopoietic differentiation potential. Optimal yield of hematopoietic cells can be achieved by expansion in mTeSR1 followed by a recovery period in MEF-CM. Furthermore, the lineage propensity to hematopoietic and neural cell types could be predicted via analysis of surrogate markers expressed by hESCs cultured in mTeSR1 versus MEF-CM, thereby circumventing laborious in vitro differentiation assays. Our study reveals that hESCs exist in a range of functional states and balance expansion with differentiation potential, which can be modulated by culture conditions in a predictive and quantitative manner. Stem Cells *2015;33:1142–1152*

## Introduction

Human embryonic stem cells (hESCs) possess the unique features of unlimited proliferation paired with an ability to differentiate into cells of all three embryonic germ layers [1–4]. Recently, hESCs have demonstrated their clinical value; they hold an enormous potential in cell replacement therapy and regenerative medicine [5]. The utility of hESCs is dependent upon our ability to generate sufficient cells of interest—a two-step process requiring pluripotent cell expansion followed by transfer to differentiation-inducing conditions. Much work has gone into understanding and optimizing the culture conditions that are required for either lineage-specific differentiation or stem cell self-renewal. However, to date, no studies have investigated how expansion-culture conditions can affect the subsequent differentiation potential of cells. Here, we demonstrate that the culture conditions of the expansion phase affect downstream lineage output of hESCs and that maximizing pluripotent cell expansion does not necessarily result in the optimum yield of differentiated cells.

Since the generation of the first hESC lines [1], technologies to develop and expand clinical grade hESCs have been innovated [6,7]. Some progress in the improvement of generation and maintenance of hESCs, such as the elimination of feeder layers and xeno-free media formulations [6,8–12], has been achieved. However, most of the developments in expansion-culture conditions have been primarily evaluated by their ability to preserve self-renewal potential [13–18]. Yet focusing research on cellular proliferation without assessing the culture's differentiation potential in subsequent differentiation conditions has been shown to lead to transformation of hESCs that possess neoplastic features [19].

By quantifying hESC expansion and subsequent differentiation to multiple lineages, we reveal that the expansion-culture formulation primes pluripotent cultures for specific lineages. This priming is reversible and can be predicted by expression levels of the surface markers, c-kit and A2B5 [20]—but not pluripotency markers. Altogether, this demonstrates that to optimize the yield of the differentiated cells of interest, the culture conditions need to be tailored to achieve the appropriate balance of cell expansion and differentiation potential.

## Materials and Methods

### Production of Mouse Embryonic Fibroblast-Conditioned Media

The primary mouse embryonic fibroblasts (MEFs) were generated from day 13, CF-1 fetuses. MEFs were expanded for two to three passages in basal MEF culture media, which is 80% of knockout Dulbecco's modified Eagle's medium (KO-DMEM) supplemented with 20% knockout serum replacement, 1 mM of l-glutamine, 1% of nonessential amino acid, and 0.1 mM of β-mercaptoethanol. Then, MEFs were irradiated and seeded with 36 × 10^6^ cells/cell stacker (636 cm^2^ growth area) on 0.1% gelatin-coated cell stacker. From next day, conditioned media have been collected and replaced with fresh MEF culture media supplemented with 4 ng/ml of hbFGF every day for consecutive 8 days. Eight days of conditioning after which the media were filtered through a 0.2 µm filter via peristaltic pump and then frozen at −80°C until use.

### hESC Culture

Three different hESC lines (H1, H9, and CA2) were maintained under feeder-free conditions [9,21,22] and passaged in either mouse embryonic fibroblast-conditioned media (MEF-CM) supplemented with 8 ng/ml of basic fibroblast growth factor (bFGF, FGF2) or mTeSR1 (Stem Cell Technologies) http://www.stemcell.com/ STEMCELL technologies, Vancouver, Canada for at least nine consecutive passages. Cells in both media conditions were passaged using 200 U/ml of collagenase IV (Life Technologies) Carlsbad, CA, USA. http://www.lifetechnologies.com/ and mechanical scoring prior to plating onto Matrigel-coated (1 ml/well of 1:15 dilution of Matrigel in KO-DMEM, overnight in the fridge) tissue culture six-well plates (BD Bioscience) http://www.bdbiosciences.com/ San Jose, CA, USA. One well of confluent ESCs in both culture media was harvested and then split into two wells (1:2 passaging ratio) every 7 days. Culture effect reversibility studies were performed by culturing hESCs for five passages in either MEF-CM or mTeSR1 followed by mTeSR1 and MEF-CM, respectively, for an additional four passages.

### Hematopoietic and Neural Differentiation of hESCs

Confluent hESC cultures at day 7 were harvested after treatment with collagenase IV to form suspension embryoid bodies (EBs) as previously described [23]. Briefly, to quantify the yield of hematopoietic and neural lineage-specific differentiation in combination with proliferation, one well of confluent hESCs (H1, H9, and CA2) in both MEF-CM and mTeSR1 media was passaged into two wells every week. One well out of two wells of confluent hESCs in each media condition had been passaged to check their proliferation and another well in each passage was used for lineage differentiation assay every passages.

Hematopoietic differentiation was carried out by culturing EBs for 20 days in 20% fetal bovine serum (FBS) containing DMEM/F-12 media supplemented with cytokines such as SCF, Flt-3L, IL-3, IL-6, G-CSF, and BMP4. EBs were then analyzed for CD45+ cell numbers by flow cytometry and for colony-forming unit (CFU) capacity in vitro. EBs differentiated without hematopoietic cytokines were also analyzed as a negative control.

For neural differentiation, EBs were generated by suspension culture in neural-proliferation media (DMEM/F-12 supplemented with 1% N2, 1% B27, 20 ng/ml epidermal growth factor, and 20 ng/ml bFGF) and cultured for 7 days with media changes at 2-day intervals. To further expand and differentiate into neural stem cells, EBs were collected and dissociated into single cells using Accutase (Sigma) http://www.sigmaaldrich.com/ St. Louis, MO, USA. Dissociated single cells were plated into low attachment six-well plates (2 × 10^4^ cells/well at passage 5 or 3 × 10^5^ cells/well at passage 5+4) with neural-proliferation media and allowed to generate neurospheres for 7 days with media changes every 3 days. At the end of each expansion and differentiation, neurospheres were collected from each well, dissociated into single cells, and counted. Neural cells were identified based on the expression of Nestin (R&D Systems) http://www.rndsystems.com/ Minneapolis, MN, USA by flow cytometry. To differentiate neural precursors along the neuronal lineage, cells were grown in DMEM/F-12 supplemented with 1× N2, 1× B27 2 µM all-trans retinoic acid (Sigma) and 5 µM forskolin (Sigma). Neural precursors were cultured in DMEM/F-12 supplemented with 1× N2, 1× B27, and 5% FBS for astrocytic differentiation. Oligodendrocyte differentiation was induced by culturing cells in DMEM/F-12, 1% N2, 1% B27, and IGF-1 (200 ng/ml).

### Hematopoietic CFU Assay

CFU assays were performed with day 20 EBs formed from hESC cultures following five passages in either MEF-CM or mTeSR1. Differentiated EBs were dissociated into single cells by the serial treatment of Collagenase B (Roche) http://lifescience.roche.com/ Roche Life Science Indianapolis, IN, USA and cell dissociation buffer (Life Technologies). Cells (1.5 × 10^3^) were then plated into methylcellulose H4230 (Stem Cell Technologies), supplemented with 25 ng/ml BMP4, 300 ng/ml SCF, 300 ng/ml Flt-3L, 10 ng/ml IL-3, 10 ng/ml IL-6, and 50 ng/ml G-CSF (R&D Systems). Hematopoietic cell clusters displaying more than approximately 50 cells were counted as colonies after incubation for 14 days at 37°C in 5% CO_2_.

### Teratoma Formation Assay

hESCs (1 × 10^6^ cells) grown in MEF-CM and mTeSR1 were injected into the testicle of immunodeficient NOD/SCID mice. Eight weeks after the injection, testicular tissues were harvested and H&E staining was performed.

### Fluorescence-Activated Cell Sorting Analysis

For undifferentiated hESC cultures, cells were dissociated with Cell Dissociation Buffer (Life Technologies) for 5–10 minutes. Dissociated single cells were incubated with SSEA3 (rat anti-human IgM, Hybridoma Bank) or A2B5 (mouse anti-human IgM, Chemicon) antibodies for 40 minutes and then identified with fluorescent-conjugated secondary antibodies (Alexa 647-conjugated goat anti-rat IgM or Alexa 647-conjugated goat anti-mouse IgM, BD Biosciences). Anti-Oct4 (BD Biosciences) staining was identified using Alexa 647-conjugated goat anti-mouse IgG (BD Biosciences). Additionally, APC-conjugated c-kit (BD Biosciences) was also used for flow. Hematopoietic or neural cells derived from day 20 EBs were detected using CD45 (BD Biosciences), nestin (R&D Systems) antibodies, respectively. Following each staining, live cells were distinguished as 7-AAD negative (BD Biosciences). Flow analysis was performed on a FACS Calibur running Cell Quest Software (BD Biosciences). Postrun analysis was performed using FlowJo version 8.5.3 (Treestar) http://www.flowjo.com/ Ashland, OR, USA.

### Image-Acquisition and Analysis

hESCs were seeded at 1 × 10^4^ cells/well (96-well imaging plate) and cultured for 5 days in either MEF-CM or mTeSR1 before fixation and permeabilization with the Cytofix/Cytoperm kit (BD Biosciences). Following blocking and washing, hESCs were immunolabeled with primary anti-SSEA3 or anti-Oct4 (BD Biosciences) and with a secondary fluorescent-conjugated antibody (Alexa Flour 488-conjugated). Nuclear staining was performed using Hoechst 33258 (Sigma). After the staining, all the images were acquired with a ×5 (0.4NA) and ×10 (0.7NA) objective on a Cellomics Arrayscan (Thermo Fisher). Images were processed and analyzed using custom scripts (Acapella 2.5., Perkin-Elmer http://www.perkinelmer.ca/ Waltham, MA, USA; ImageJ, NIH) http://imagej.nih.gov/ij/.

hESC-derived astrocytes, neurons, and oligodendrocytes were characterized by immunocytochemical staining with glial fibrillary acidic protein (GFAP), Tuj1, and O4 antibodies (Santa Cruz) Santa Cruz Biotechnology Inc. http://www.scbt.com/ Santa Cruz, CA, USA, respectively. Image Pro (Media Cybernetics) http://www.mediacy.com/ Rockville, MD, USA was used for morphometrical analysis.

hESCs seeded at 5 × 10^4^ cells/well in 24-well plates were cultured for 5 days in either MEF-CM or mTeSR1 and incubated in a Nikon BioStation CT allowing automated image acquisition of the cultures. R-Phycoerythrin-conjugated Annexin V (Life Technologies) was added to the media (1:1,000) 24 hours postseeding and the median dye exchanged each day. Phase contrast and fluorescent images of manually selected colonies were acquired every 4 hours at ×2 for quantification and ×10 for visualization. Images were processed and analyzed as described above.

### Gene Expression Analysis

Total RNA was extracted (Norgen Biotek) and hybridized to the Affymetrix Gene Chip Human Gene 1.0 ST arrays (London Regional Genomics Centre, ON, Canada). Output data were normalized using robust multichip averaging algorithm and baseline transformation to the median of all samples using GeneSpring 12.0 software (Agilent Technologies). Normalized arrays were hierarchically clustered based on differentially expressed gene list with *p* ≤ .05 and fold change ≥5, which were subsequently compared using Pearson's correlation coefficient in order to generate dendograms. Multiple hypotheses testing such as Benjamini-Hochberg false discovery rate *p*-value correction was performed for comparison. Scatter plots were generated using all entities.

Gene set enrichment analysis (GSEA) was performed on normalized expression values of all entities between samples using GSEA software v2.07 (Broad Institute). Curated gene sets such as Nervous System Development (M7312) and Kegg Hematopoietic Cell Lineage (M6856) from Molecular Signatures Database (MSigDB) were used to find enrichment.

### Statistical Analysis

Student's *t* test was applied for statistical analyses. Error bars denote SD through this study.

## Results

### Expansion-Media Composition Introduces Lineage Bias in Subsequent Differentiation Assays

Previously our laboratory has optimized the conditions to derive functional hematopoietic cells from hESC cultures [24–26]. In this study, we have performed a side-by-side comparison of hESC production in MEF-CM and the commercially available, defined media mTeSR1 [27] and their subsequent differentiation. Three separate hESC lines (CA2, H9, and H1) adapted in MEF-CM were switched to mTeSR1 or continued in MEF-CM.

All experimental results were based on three biological sources of hESCs (H9, H1, and CA2) and independent experiments representing different passage numbers were used for each cell line for a total of six or more repeats (four repeats with H9); three cell lines × two experiments for each = six. This approach was used to assure the study supports generalizable effects on hESCs, rather than effects limited to individual cell lines or dependence on passage numbers specific behavior.

**Figure 1:**
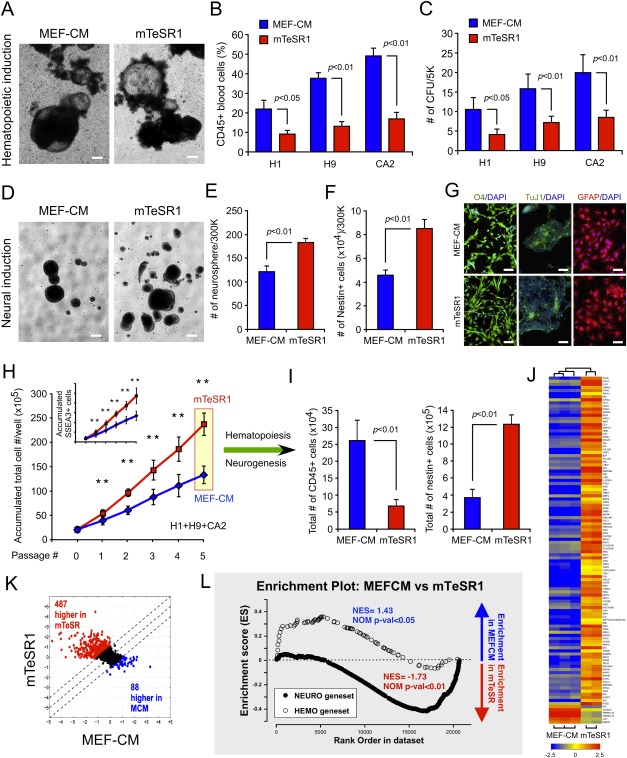
Lineage-specific differentiation and gene signatures of human embryonic stem cells (hESCs) can be controlled by culture media. (A–C): Hematopoietic differentiation of three different hESC (H1, H9, and CA2) lines at passage 3 in the indicated media. Apart from morphological similarities (A), EBs formed with MEF-CM-hESCs consistently showed higher frequencies of CD45+ blood (B), and hematopoietic CFUs (C) compared to mTeSR1-hESCs. Error bars denote SD. *n* = 6 (*n* = 2/cell line). Scale bars = 100 µm. (D–G): Neural differentiation potential is augmented in mTeSR1-hESCs. With 300,000 seeding for neurosphere assay (D) at passage 3, higher frequencies of neurospheres (E) and Nestin+ cells (F) were found in mTeSR1-hESCs. Cells in neurospheres from both conditions have similar potential to be specified into oligodendrocytes (O4+), neurons (Tuj1+), and glial cells (GFAP+) (G). H1, H9, and CA2 were tested (*n* = 6, *n* = 2/cell line). Error bars represent SD. Scale bars = 100 µm. (H, I): Quantitative measurements of hematopoietic and neural differentiation from three hESC lines after five passages in the indicated media (*n* = 8; *n* = 2/H1 and CA2, *n* = 4/H9). A roughly twofold increase of accumulated total (H) and SSEA3+ cells (H; inset graph) in mTeSR1-hESCs is observed. Total number of Nestin+ cells was significantly increased whereas total number of CD45+ blood cells was decreased in mTeSR1-hESCs (I). **, *p* < .01. Error bars denote SD. (J–L): Global gene expression was modulated by hESC expansion media. Dendogram with heat map (*p* ≤ .05 and more than or equal to fivefold change, J) and unfiltered scatter plot (K) shows a higher number of genes expressed in mTeSR1 (*n* = 2) cultures compared to MEF-CM (*n* = 3) expanded hESCs. Enrichment plot represented neuronal development genes that are highly expressed in mTeSR1-hESCs whereas hematopoietic genes are highly expressed in MEF-CM-hESCs (L). Abbreviation: MEF-CM, mouse embryonic fibroblast-conditioned media.

A consequence of expansion in mTeSR1 was a change in the differentiation capabilities of the cells. MEF-CM cultured hESCs were transferred to mTeSR1 media prior to quantification of differentiation potential. hESCs expanded in mTeSR1 media for three consecutive passages partially lost their ability to differentiate toward the hematopoietic lineage in subsequent hematopoietic embryoid body (EB) assays. Despite no difference in the morphology of EBs generated using cells expanded in either mTeSR1 or control MEF-CM (Fig. 1A), the frequency of cells expressing blood-specific CD45 (Fig. 1B) and levels of hematopoietic progenitors, which were quantified by CFU assay (Fig. 1C), were reduced by approximately threefold in mTeSR1 compared with MEF-CM expanded cells.

Next, neural lineage differentiation was assessed through the generation of neurospheres (Fig. 1D). hESCs expanded for three continuous passages in mTeSR1 produced a greater number of neurospheres (Fig. 1E) and Nestin+ cells (Fig. 1F) than hESCs expanded in MEF-CM. Neural precursors within neurospheres from both media conditions had similar specification potential yielding oligodendrocytes (O4+), neurons (Tuj1+), and glial cells (GFAP+) (Fig. 1G). These data suggested that hematopoietic and neural differentiation potentials could be controlled by culture media for undifferentiated hESCs.

### Behavioral Changes of hESCs Accompanied by Gene Signature Alterations

Upon qualitative assays for lineage-specific differentiation at passage 3, quantitative measurement of hematopoietic and neural output combined with altered proliferation was performed at passage 5. The neural-priming, coupled to the twofold increase in expansion of total (Fig. 1H; red box) as well as SSEA3+ (Fig. 1H; inset graph) cells which have different cellular features (Supporting Information Fig. S1A–S1J), resulted in the generation of greater number of total neural cells in mTeSR1 than MEF-CM. Conversely, mTeSR1 expanded hESCs impaired in CD45+ blood cell production (Fig. 1I).

Surprisingly, gene signature has been regulated upon hESC media change (Fig. 1J–1L). Global gene expression analysis revealed that growth of hESCs in mTeSR1 augmented global transcription levels relative to MEF-CM by heat map (more than or equal to fivefold change) (Fig. 1J; Supporting Information Table S1) and scatter plot (Fig. 1K). mTeSR1 cultures resulted in an increase in expression of 487 genes and decrease in only 88 (Fig. 1K). Additionally, enrichment of lineage-specific genesets correlated with lineage differentiation potential with MEF-CM and mTeSR1 expanded cultures showing enhanced hematopoietic and neural gene signatures, respectively (Fig. 1L). These data suggested that hematopoietic and neural differentiation potential of hESCs can be primed toward specific lineages in subsequent differentiation conditions by initial culture media conditions.

### Lineage-Priming Can Be Reversed by Culture Media

**Figure 2:**
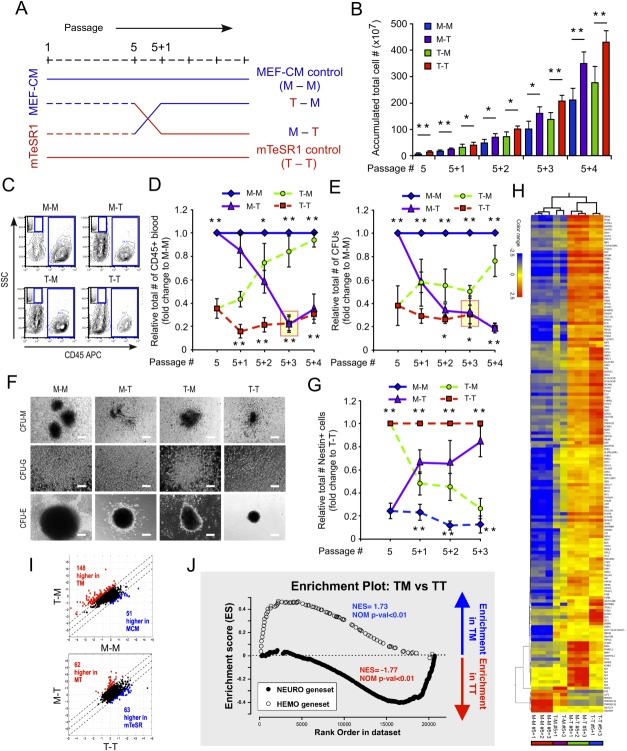
Reversible modulation of human embryonic stem cell (hESC) proliferation, lineage-specific differentiation, and gene signatures by switching culture media. (A): Experimental schematic to examine culture-mediated reversibility of growth and differentiation potential of hESCs. Proliferation and lineage-specific differentiation assays were performed over four passages after media switch with hESCs, which were continuously expanded in MEF-CM (M-M) and mTeSR1 (T-T) throughout as well as newly adapted in mTeSR1 (M-T) and MEF-CM (T-M) from MEF-CM and mTeSR1, respectively. H1, H9, and CA2 were used through this study (*n* = 8; *n* = 2/H1 and CA2, *n* = 4/H9). Error bars denote SD. (B): hESCs newly adapted to mTeSR1 (M-T) showed higher accumulated total cell numbers relative to hESCs maintained in MEF-CM throughout (M-M), whereas cells transferred to MEF-CM (T-M) underwent a gradual reduction in accumulated total cell numbers compared to mTeSR1 controls (T-T). *, *p* < .05; **, *p* < .01. (C–F): Changes in blood differentiation potential were monitored at each passage for four passages after media switch. Flow cytograms acquired two passages after media switch displayed reduced and increased CD45 expressions in M-T and T-M conditions compared to M-M and T-T, respectively (C). Gradual change of CD45+ blood emergence in relative total blood numbers showed a decrease in mTeSR1 versus a reversion to augmented blood differentiation potential for cells transferred to MEF-CM (D). Similarly, MEF-CM expanded hESCs generated higher numbers of CFUs, which decreased after switching to mTeSR1; whereas mTeSR1 exchanged to MEF-CM induced a recovery of accumulated CFU numbers over four passages (E). However, similar blood subtypes were observed among all media conditions (F). *, *p* < .05; **, *p* < .01. (G): Relative total number of Nestin+ cells showed higher neural differentiation was achieved from hESCs expanded in mTeSR1 (T-T) than MEF-CM media condition (M-M). Immediately after switch hESC culture media from MEF-CM to mTeSR1 (M-T), the number of Nestin+ cells was rapidly increased and was similar to that in T-T. Conversely, the number of Nestin+ cell was continuously reduced to that in MEF-CM throughout. *, *p* < .05; **, *p* < .01. (H, I): Global gene expression profile of hESCs was modulated by culture media in a reversible manner. Dendogram with heat map (*p* ≤ .05 and more than or equal to fivefold change, H) and unfiltered scatter plots (I) represented reversible gene expression regulation by changing culture media. (J): Expression of lineage-specific genes was reversed by switching culture media. Enrichment plots showed hematopoietic genes were preferentially expressed in T-M (*n* = 2) condition whereas neural genes were active in T-T (*n* = 2). Abbreviation: MEF-CM, mouse embryonic fibroblast-conditioned media.

One outstanding question is whether priming toward a particular lineage is an irreversible commitment: irreversibility would point to a simple clonal selection; reversibility could provide a potential mechanism of modulating differentiated cell output. We switched MEF-CM- and mTeSR1-expanded cells back to mTeSR1 and MEF-CM, respectively (Fig. 2A) then quantified the changes in hematopoietic and neural differentiation over a period of four passages.

As was seen previously (Fig. 1H) human PSCs switched from MEF-CM to mTeSR1 (M-T) rapidly increased total cell numbers in comparison to those continuously grown in MEF-CM (M-M). This change was significant (*p* < .01) after even a single passage (Fig. 2B). Conversely, hESCs that were readapted to MEF-CM from mTeSR1 (T-M) slowed down their proliferation compared to hESCs grown in mTeSR1 throughout (T-T) (Fig. 2B) and their morphology reverted to that of M-M cultures (Supporting Information Fig. S2A). The frequency of SSEA3+ cells remained consistent across all cultures at between 45% and 50% indicating no change in the pluripotent cell compartment (Supporting Information Fig. S2B, S2C). However, accumulated SSEA3+ cell numbers were increased in mTeSR1 media (M-T) whereas were decreased in MEF-CM (T-M) (Supporting Information Fig. S2D, S2E).

After four passages back in MEF-CM, the hematopoietic potential of mTeSR1 expanded cells recovered to that of M-M expanded cells. Switching from mTeSR1 to MEF-CM (T-M) resulted in a steady recovery in the frequency and accumulated number of CD45+ cells (Fig. 2C, 2D and Supporting Information Fig. S3A) and CFUs (Fig. 2E, 2F and Supporting Information Fig. S3B) in hematopoietic differentiation assays. The reduction in CD45+ and CFU potential seen previously in MEF-CM expanded cells three passages after switching to mTeSR1 (Fig. 1B, 1C) actually started after a single passage in mTeSR1—reaching a plateau at passage 3 (Fig. 2D, 2E; red boxes). The frequency of progenitor differentiation, measured by CFU assay, seemed slower to recover than that of CD45+ cell differentiation (Fig. 2D, 2E; green lines). Despite differences in the number of CFU colonies, the colonies across cultures showed similar morphology (Fig. 2F) and frequency (Supporting Information Fig. S3C) of blood subtypes (CFU-G, -M, -E, -GM, and -Mix) in methylcellulose.

While switching media from mTeSR1 to MEF-CM (T-M) augmented blood differentiation capacity, it simultaneously diminished the neural differentiation potential of hESCs (Fig. 2G and Supporting Information Fig. S3D). Again, this change occurred rapidly after a single passage in MEF-CM, reaching the same frequency of cultures maintained in MEF-CM throughout after three passages. Similarly in the reverse experiment, cells newly adapted to mTeSR1 from MEF-CM (M-T) demonstrated augmented neural differentiation capacity compared to hESCs grown in MEF-CM throughout (M-M). This augmentation occurred after a single passage in mTeSR1 and the frequency (Fig. 2G) and accumulated number (Supporting Information Fig. S3D) of Nestin+ cells increased to maximum levels after three passages.

Consistent with reversible modulation of lineage-specific differentiation, hESC-media conditions also altered global gene expression profiles in a reversible manner (Fig. 2H–2J). Both hierarchical clustering of genes showing greater than fivefold difference in expression values (Fig. 2H; Supporting Information Table S2) and global comparison of expression data (Fig. 2I) revealed that gene signatures of hESCs switched to mTeSR1 or MEF-CM are most similar to cells grown under these conditions throughout, and are thus reversible. Further to this, blood-specific genesets were enriched in T-M conditions (Fig. 2J and Supporting Information Fig. S4A), consistent with the observed augmented blood formation potential (Fig. 2C–2E); whereas neural-specific genesets were enriched in M-T (Fig. 2J and Supporting Information Fig. S4B). These observations suggest that media-induced lineage-specific gene expression alteration is reversible along with differentiation capacity of hESCs. In summary, these data show for the first time that the differentiation potential of hESCs can be reversibly modulated in vitro by expansion culture conditions prior to the differentiation treatment regime.

### Expansion Media Control hESC Pluripotency State In Vitro But Not In Vivo

**Figure 3:**
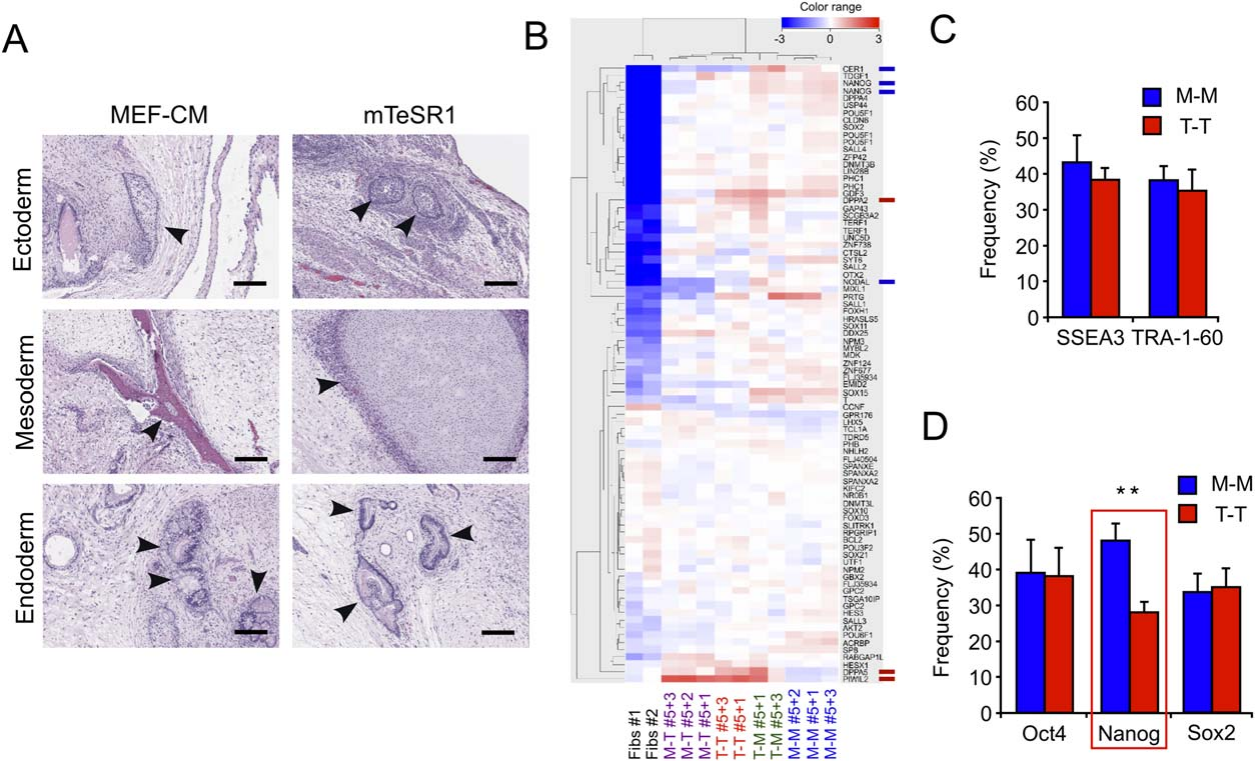
Culture media does not change in vivo differentiation potential but rebalances pluripotency states of human embryonic stem cells (hESCs) in vitro. (A, B): hESCs expanded in either MEF-CM (*n* = 3) or mTeSR1 (*n* = 3) demonstrate the ability to form all three embryonic germ layers in vivo (teratoma formation, A). However, a subset of pluripotency-associated genes was more highly expressed in cells grown in MEF-CM (B). Red bars, genes enriched in mTeSR1-hESCs; blue bars, genes enriched in MEF-CM-hESCs. (C, D): Expressions of pluripotency-associated cell-surface (C) and intracytoplasmic (D) markers by fluorescence-activated cell sorting (*n* = 8; *n* = 2/H1 and CA2, *n* = 4/H9). The expression levels of SSEA3, TRA-1–60 (C), Oct4, and Sox2 were similar in MEF-CM-(M-M) or mTeSR1 (T-T) expanded hESCs. However, Nanog expression level was significantly higher in MEF-CM expanded hESCs than mTeSR1 expanded. **, *p* < .01. Error bars denote SD. Abbreviation: MEF-CM, mouse embryonic fibroblast-conditioned media. M-M = cells cultured for 5 passages in MEFCM; T-T = cells cultures in five passages of mTeSR.

By performing quantitative assays in vitro, we have described unique features of hESCs, which are proliferation and their subsequent lineage differentiation, can be controlled by media conditions in a reversible manner. To determine whether hESC expansion media also modulate pluripotency in vivo, the qualitative teratoma formation assay was performed and demonstrated that cells grown in both MEF-CM and mTeSR1 are able to form teratomas containing cells of all three germ layers (Fig. 3A). However, rebalancing pluripotency states was further investigated by analyzing the expression of transcripts (Fig. 3B) and proteins (Fig. 3C, 3D) since pluripotency states of hESCs were regulated by expansion media conditions in vitro. Expression levels of genes found to be enriched across a range of hESC lines [2] were compared between MEF-CM- and mTeSR1-expanded hESCs. Of the 97 genes in the pluripotency geneset, only 2 (DPPA5 and PIWIL2) showed a greater than twofold change in expression in mTeSR1-expanded hESCs (Fig. 3B). PIWIL2, which is one of the genes upregulated (4.8-fold) in mTeSR1 compared to MEF-CM hESC cultures, is associated with suppression of apoptosis in cancer stem cells and may underpin the reduced apoptosis seen in mTeSR1-grown cultures [28,29]. Conversely, MEF-CM hESC cultures, which possess higher blood differentiation potential, showed higher Nanog expression levels in both transcript (Fig. 3B) and protein (Fig. 3D). Overall, there was no concomitant change in the frequency of cells positive for pluripotency markers by fluorescence-activated cell sorting (FACS) analysis (Fig. 3C, 3D) and immunocytochemical staining (Supporting Information Fig. S5) even though total cell numbers produced in mTeSR1 media condition were nearly doubled compared to MEF-CM (Fig. 2B): SSEA3, TRA-1–60 (surface markers, Fig. 3C), Oct4, and Sox2 (intracytoplasmic markers, Fig. 3D). However, another of the core pluripotency markers, Nanog, was more highly expressed in MEF-CM condition, which have enhanced hematopoietic differentiation potential (Fig. 3D; red box).

### Differentiation Potential Can Be Predicted by the A2B5 and c-Kit Surface Markers

Although changes in global gene expression profiles were correlated with lineage-bias, it is desirable to have a more practical metric of culture differentiation potential prior to lengthy, costly differentiation assessments. Previously, we have demonstrated that the expression levels of c-kit and A2B5 in undifferentiated hESCs in vitro can be used in a predictive manner to gauge blood and neural differentiation-potential in subpopulations of hESCs from MEF-CM expanded cultures [20]. We hypothesized that the similar lineage biases seen in mTeSR1 and MEF-CM expanded cells may correspond to a shift in these subpopulations and provide a useful tool to quantify the differentiation potential of hESC cultures.

**Figure 4:**
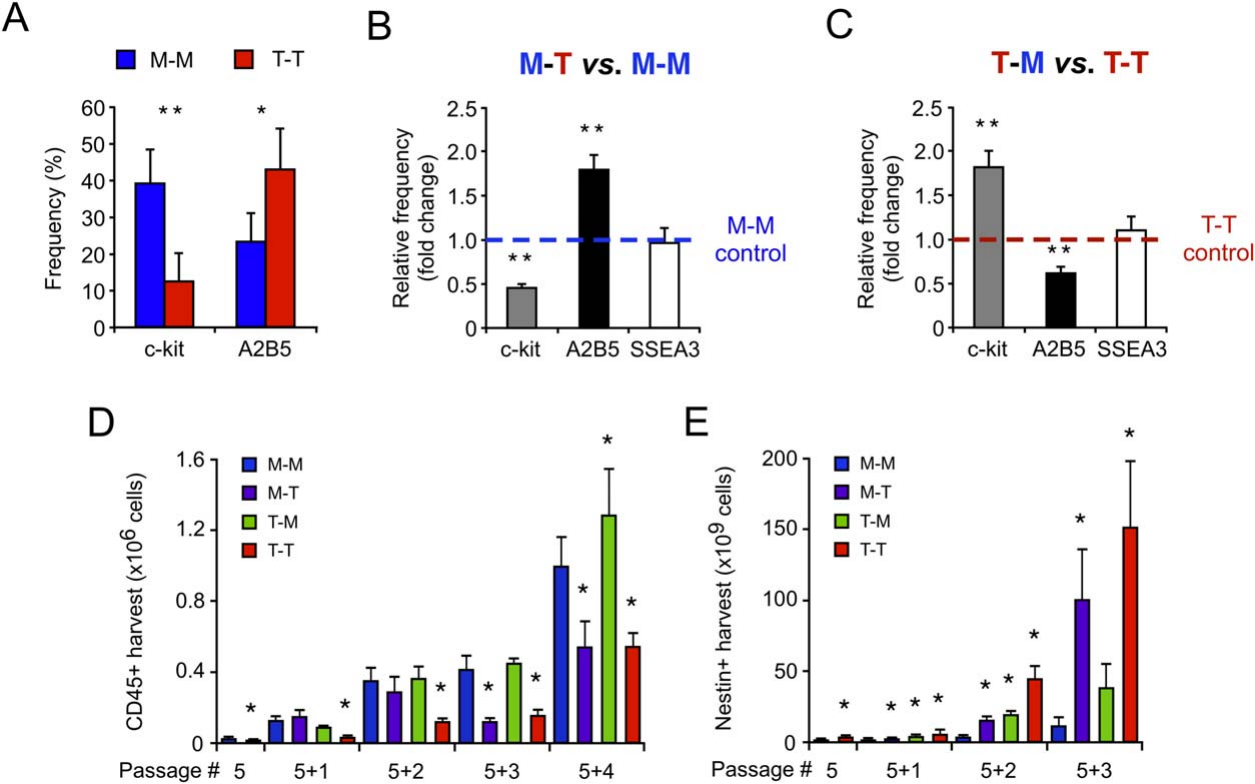
Lineage-specific surrogate markers enable to predict subsequent in vitro lineage-specific differentiation of human embryonic stem cells (hESCs). (A–C): Expression level of the predictive hematopoietic marker, c-kit, is significantly lower in mTeSR1- versus mouse embryonic fibroblast-conditioned media (MEF-CM)-hESCs whereas A2B5 expression was greater in mTeSR1-hESCs (A). Fluorescence-activated cell sorting analysis revealed differential expression of lineage-specific markers in undifferentiated hESCs at passage #5+4. hESCs switch from MEF-CM to mTeSR1 (M-T) show higher ectoderm-specific A2B5 expression and lower c-kit expression in relation to MEF-CM (M-M) control (B). Conversely, T-M conditions increased c-kit and decreased A2B5 expression compared to mTeSR1 (T-T) controls (C). H1, H9, and CA2 were analyzed (*n* = 8; *n* = 2/H1 and CA2, *n* = 4/H9). **, *p* < .01; *, *p* < .05. (D, E): Lineage-priming by undifferentiated hESC expansion cultures controlled hematopoietic (D) and neural (E) cell production yield. Four passages after expansion media switch from mTeSR1 to MEF-CM (T-M), blood production yield is much higher than original MEF-CM (D). Meanwhile, cells in mTeSR1 throughout maximized neural production yield compared to other conditions (E). Differentiated cell production yield from other expansion conditions was compared to MEF-CM condition. H1, H9, and CA2 were analyzed (*n* = 8; *n* = 2/H1 and CA2, *n* = 4/H9). *, *p* < .05.

mTeSR1 expanded cultures had a minimal levels of c-kit+ cells (<15%), while cells maintained in MEF-CM showed a c-kit+ fraction of nearly 40% (Fig. 4A). In contrast, the expression of the neural precursor marker A2B5 was significantly higher in mTeSR1 cultures when compared with those maintained in MEF-CM (Fig. 4A). The correlation of these data and the skewed differentiation potential of MEF-CM and mTeSR1 cultures toward the hematopoietic and neural lineages, respectively, validate the use of c-kit and A2B5 as early surrogate markers of hESC differentiation potential.

Furthermore, these markers also have utility in quantifying the recovery of differentiation potential upon media switching. Following five passages in either MEF-CM or mTeSR1, cells switched from MEF-CM to mTeSR1 (M-T) showed a significant decrease in the relative expression of c-kit and a greater level of expression of A2B5 in relation to MEF-CM (M-M) controls (Fig. 4B). Conversely, hESCs cultured first in mTeSR1 and then switched to MEF-CM (T-M) had augmented c-kit levels and decreased A2B5 expression compared to mTeSR1 (T-T) controls (Fig. 4C). These findings support the use of A2B5 and c-kit as predictors of differentiation potential.

### Optimized Lineage Specification Requires Balancing Expansion and Differentiation

The yield of differentiated cells is the product of initial cell number and frequency of differentiation. Since initial expansion in mTeSR1 resulted in twofold more pluripotent cells than MEF-CM expanded cells (Fig. 1H), the theoretical yield of differentiated cells at each passage was calculated.

The reversibility of the lineage-priming does provide a mechanism by which expansion and hematopoietic lineage-potential can be tuned. The combination of increased expansion (Fig. 1H) and full recovery of hematopoietic potential (Fig. 2D, 2E) means that optimal yield of CD45+ cells was obtained from only five passages in mTeSR1 before transfer to MEF-CM for four passages (Fig. 4D). Longer expansion in mTeSR1 prior to returning to MEF-CM for four passages would be predicted to further increase the yield above MEF-CM continually expanded cells.

The increase in cell expansion coupled with the increase neural lineage-priming means that switching MEF-CM grown cells to mTeSR1 results in an immediate increase in yield of Nestin+ cells in subsequent differentiation assays (Fig. 4E). However, this increase in pluripotent cell yield in mTeSR1 did not offset the reduction in hematopoietic potential resulting in a reduction in CD45+ harvest (Fig. 4D) after only three passages in mTeSR1.

**Figure 5:**
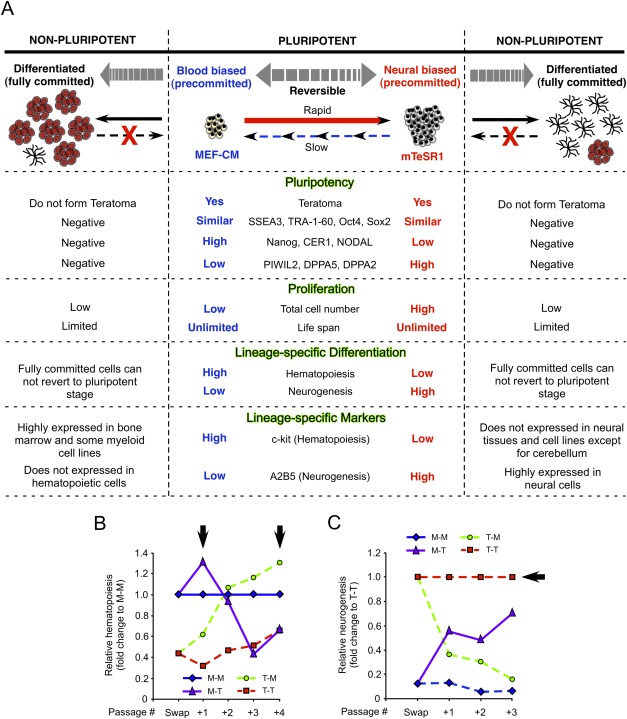
Balancing proliferation and differentiation potential of human embryonic stem cells (hESCs) and the use of surrogate markers to predict differentiation potential. (A): Summary of reversible modulation of hESC proliferation and lineage-specific differentiation by regulating pluripotency states upon expansion media conditions. (B, C): Optimal combinations of media conditions and passage numbers to produce maximum numbers of hESC-derived hematopoietic (B) and neural (C) cells. Black arrows indicated optimal lineage-priming conditions for hematopoietic (B) and neural (C) differentiation of hESCs. Abbreviation: MEF-CM, mouse embryonic fibroblast-conditioned media.

Overall summary demonstrates that lineage differentiation coupled with proliferation activity is primed by rebalancing pluripotency states (Supporting Information Fig. S4C) upon media conditions for hESC expansion. Additionally, lineage-specific cell surface markers enable to predict lineage specification of hESCs (Fig. 5A) even though the expression level of hematopoietic or neural lineage-specific genes is quite low in undifferentiated hESCs compared to fully mature types of cells (Supporting Information Fig. S6A, S6B).

Through the quantitative measurement of pluripotent cell expansion along with blood and neural differentiation, we can propose optimized media conditions for blood (Fig. 5B) and neural (Fig. 5C) differentiation. For instance, a 1.3-fold increase in blood cells can be attained using hESCs at passage #1 and #4 in M-T and T-M following media switching, respectively. This is seen as a balance between total cell numbers, which are augmented by culture in mTeSR1, and increased blood cell frequencies, which are highest in MEF-CM. With respect to neural differentiation, no other condition is better than maintenance in mTeSR1 throughout.

## Discussion

Herein, we have demonstrated, for the first time, that hESCs are capable of undergoing reversible changes in early cell fate decisions in response to culture conditions prior to their culture in differentiation-inducing media. In contrast to MEF-CM cultures, mTeSR1 grown cultures are characterized by their high proliferation rate, lower hematopoietic potential, and higher neural potential (Fig. 5A). To date, selection of hESC media has been heavily centered on expansion of embryonic stem cells for clinical translational purposes. This article highlights the need for independent assessment of proliferation and differentiation capacity.

Our findings demonstrate that the yield of neural cells from standard neural-inducing conditions can be increased if the hESCs are expanded in mTeSR1. However, this increase in the frequency of neural differentiation is at the expense of hematopoietic differentiation. This lineage-bias could be modulated in a reversible manner within a fairly short timeframe. By focusing the effect of expansion-media on downstream differentiation we have highlighted the importance of quantifying differentiation when expanding hESCs. Interestingly, this dynamic reversible modulation of hESC proliferation and lineage-specific differentiation was consistently found in fibroblast-derived induced pluripotent stem cells (iPSCs, data not shown) upon media condition. We believe that comparative studies of general features of hESCs and iPSCs with regard to similarity and difference could provide useful information for the potential use of hESCs and/or iPSCs in the field of regenerative medicine [30].

Several publications have focused on the role that the culture environment plays on hESC propagation and differentiation. Particularly, certain recent studies have also observed that cells cultured in mTeSR1 displayed enhanced propagation versus cells grown in MEF-CM [13,31]. However, the depth of this augmentation and nature of its reversibility have not been described previously. In addition to these studies, another recent paper has shown that mTeSR1 cultures are less efficient at differentiation toward the cardiac lineage (another mesodermal cell type such as blood) when compared with MEF-CM cultures; noting that mTeSR1 cultures had enhanced neural characteristics [32]. Such lineage-priming has been traditionally viewed as the first step toward lineage commitment with cells progressively extinguishing expression of pluripotency associated genes with concomitant activation of lineage-specific transcripts. Our work indicates instead that lineage-priming should be viewed as a reversible property of hESCs directly influenced by their culture environment. The mechanism behind this priming is unclear but the reversibility indicates that it is not based on clonal selection. Previously we have highlighted the role of the stem cell generated niche in modulating differentiation [33,34]. Changes in the nonpluripotent niche, which makes hESC culture heterogeneous, would be consistent with our observations that pluripotent markers remained largely unaffected by mTeSR1 (Supporting Information Fig. S5).

The promised medical advances associated with stem cells will only be possible if we can achieve large-scale generation of functional cells of interest. Optimally this would be achieved by rapid expansion of pluripotent cultures followed by efficient differentiation of these cultures to differentiated cells of the desired lineage. Counterintuitively, the maximum generation of differentiated cells of the desired lineage may not be achieved with conditions resulting in the greatest pluripotent cell production. However, the reversibility of lineage-priming means that tailoring the expansion conditions across multiple passages can result in optimized generation of differentiated cells of interest. In our hands, expansion of hESCs in mTeSR1 for four passages followed by readaptation to MEF-CM for another five passages resulted in an improved yield in blood cells. A longer expansion phase in mTeSR1 would be predicted to further improve hematopoietic-yield over cells continuously cultured in MEF-CM alone.

Early prediction of hESC differentiation potential is paramount to the proper evaluation of culture systems with respect to scalability and downstream assays. Quantification of pluripotency markers and transcription factors such as SSEA3, TRA-1–60, Oct4, and Sox2 failed to identify the shift in the differentiation potential of the culture. However, surrogate markers of early neural and hematopoietic differentiation, namely A2B5 and c-kit [20], as well as one of pluripotent markers Nanog were. Our previous study revealed that surrogate markers facilitate efficient lineage-specific differentiation by performing downstream differentiation assay with A2B5 or c-kit positive subset [20]. Nanog's higher expression in MEF-CM may itself be predictive of an enhanced mesodermal signature given its proangiogenic properties [35]. The ability of specific culture media to dictate hESC priming toward the hematopoietic or neural lineage illustrates the heterogeneous and dynamic nature of the embryonic stem cell state while delineating studies that must be performed for accurate characterization of culture systems.

## Conclusions

In summary, we demonstrate that heterogeneity of hESCs extends to functional characteristics that can be reversibly modulated by culture conditions, illustrating the importance of the choice of culture conditions for regenerative medicine or drug discovery applications.
