# Supplementary figures and images for: Reversible Lineage-Specific Priming of Human Embryonic Stem Cells Can Be Exploited to Optimize the Yield of Differentiated Cells
Source: Stem Cells. 2015 Jan 13;33(4):1142–52. doi: 10.1002/stem.1952 (PMC4413029; doi:10.1002/stem.1952)

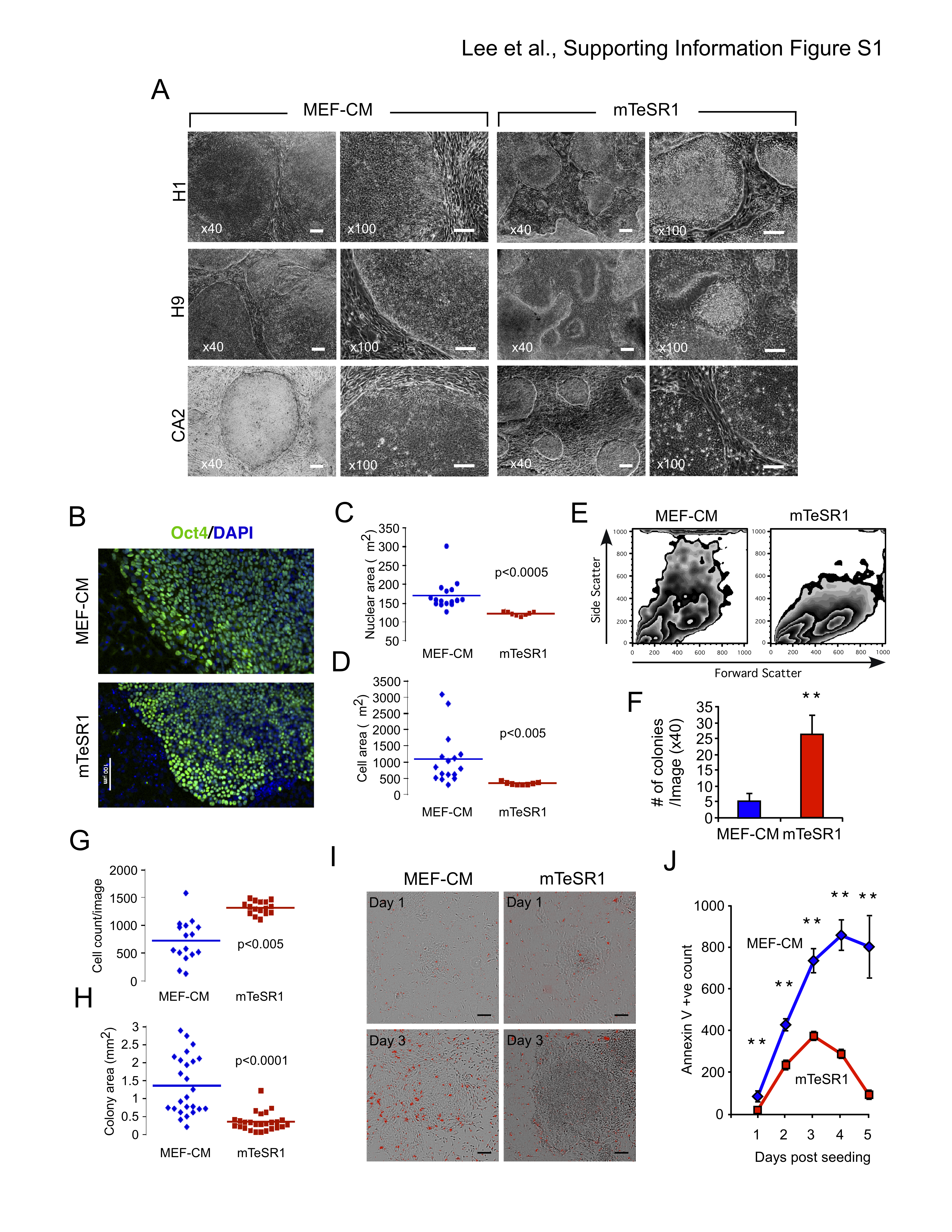

Supplement: Supplementary file 1 — Supporting Figure 1 [file stem0033-1142-sd1.tiff]

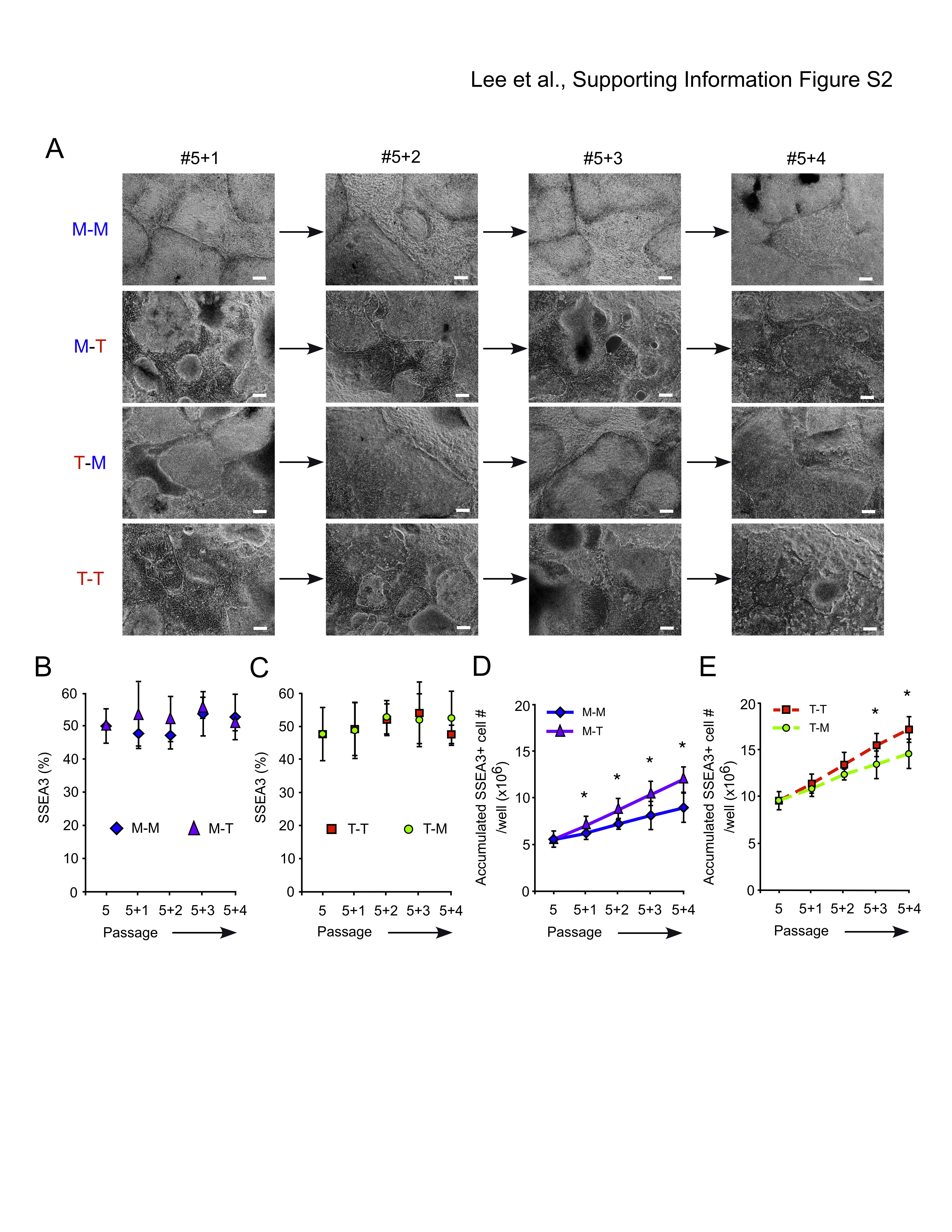

Supplement: Supplementary file 2 — Supporting Figure 2 [file stem0033-1142-sd2.tiff]

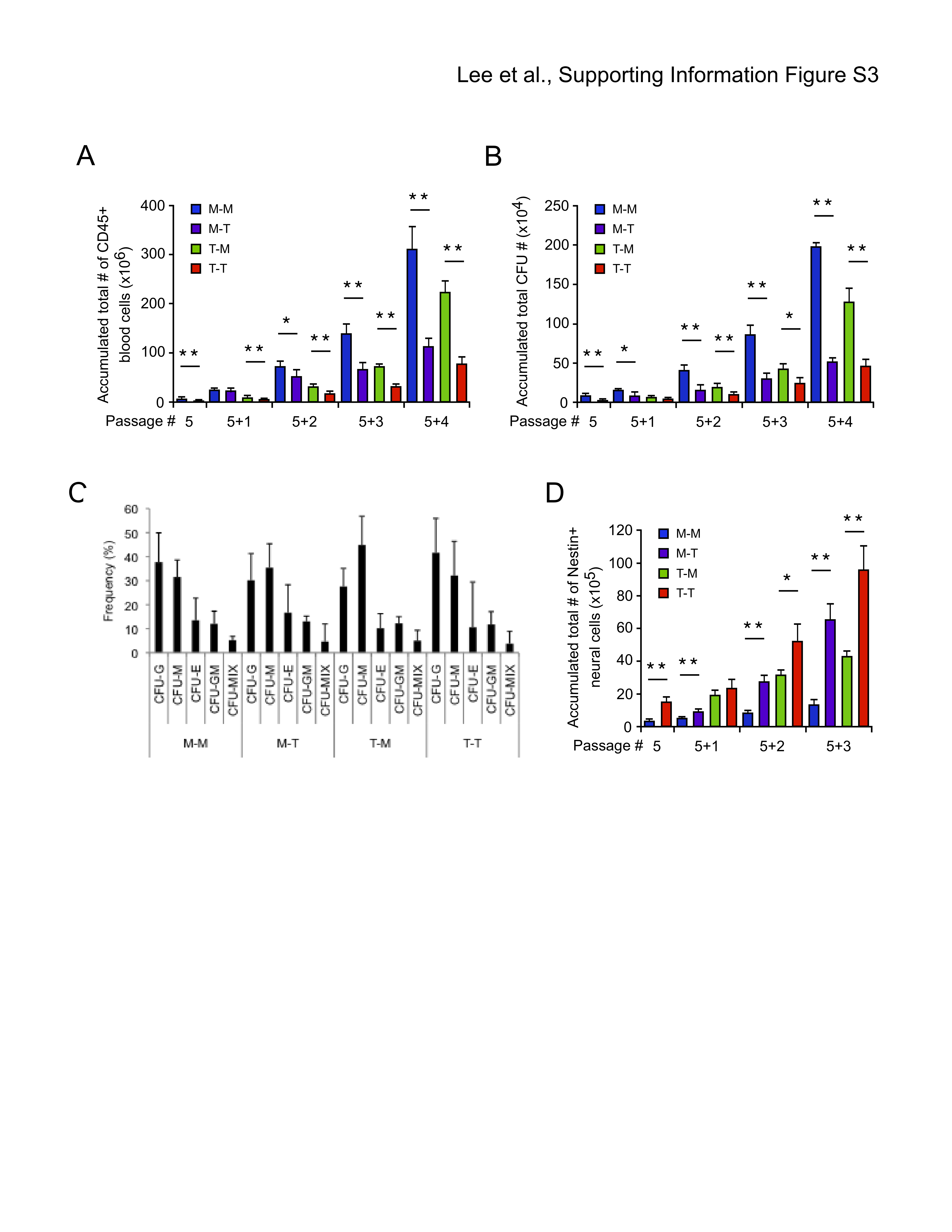

Supplement: Supplementary file 3 — Supporting Figure 3 [file stem0033-1142-sd3.tiff]

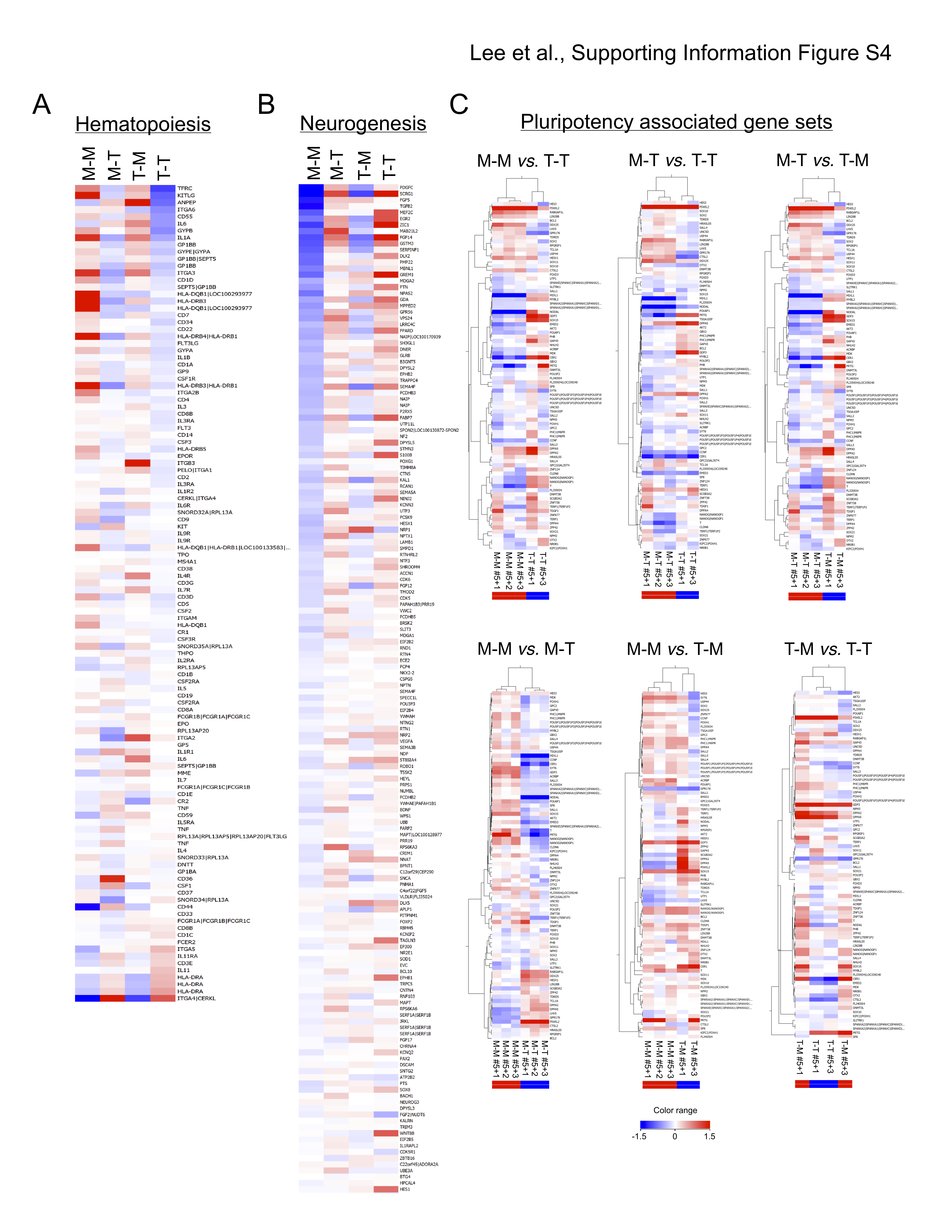

Supplement: Supplementary file 4 — Supporting Figure 4 [file stem0033-1142-sd4.tiff]

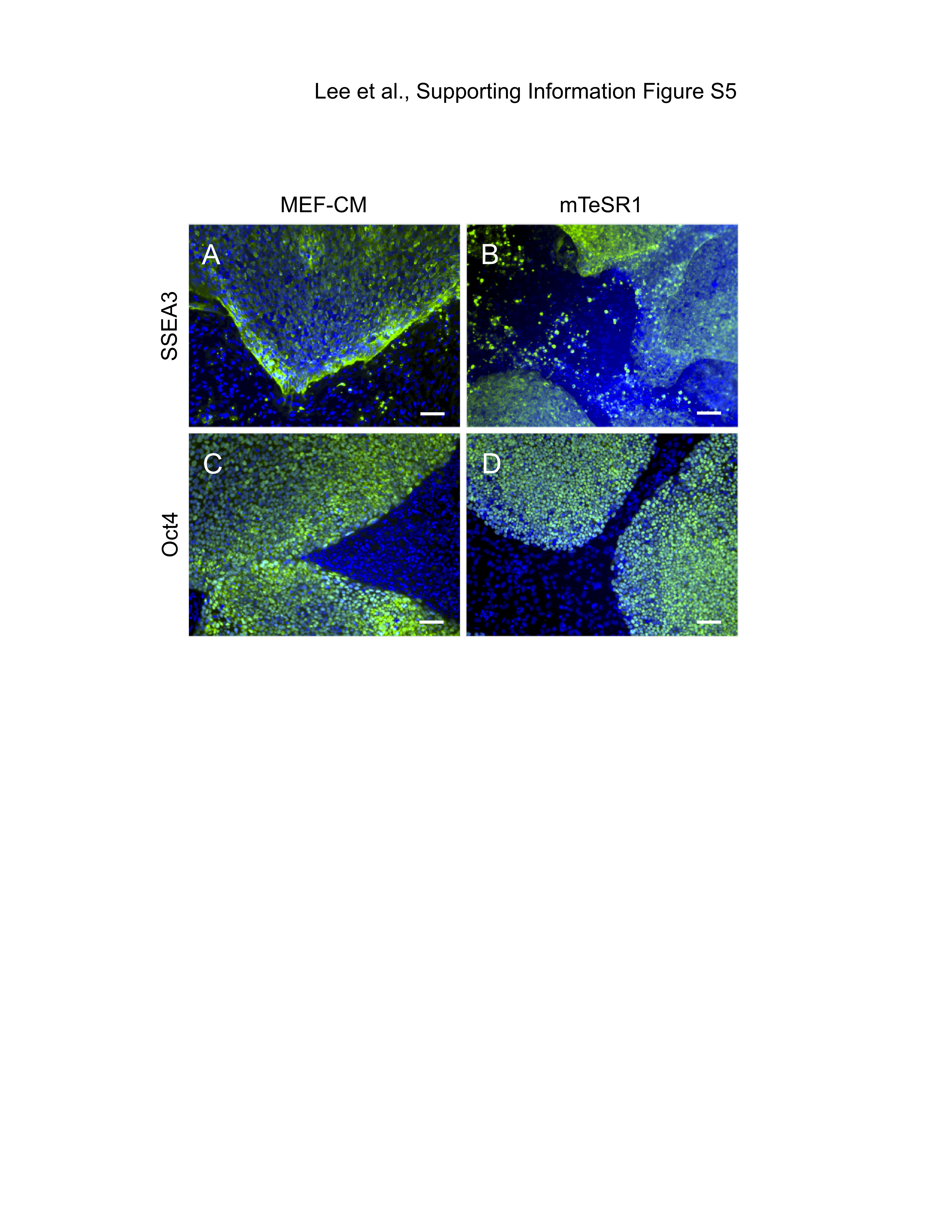

Supplement: Supplementary file 5 — Supporting Figure 5 [file stem0033-1142-sd5.tiff]

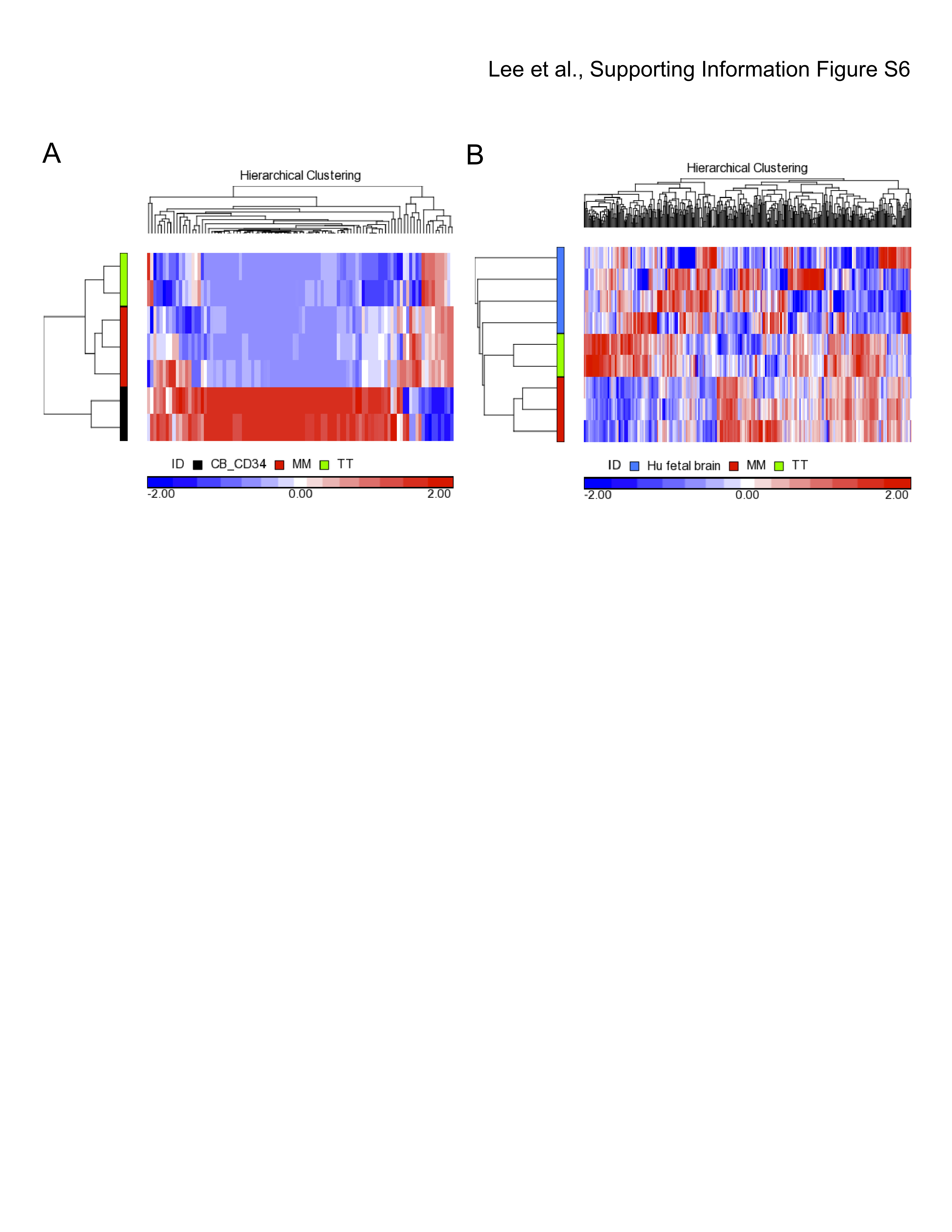

Supplement: Supplementary file 6 — Supporting Figure 6 [file stem0033-1142-sd6.tiff]

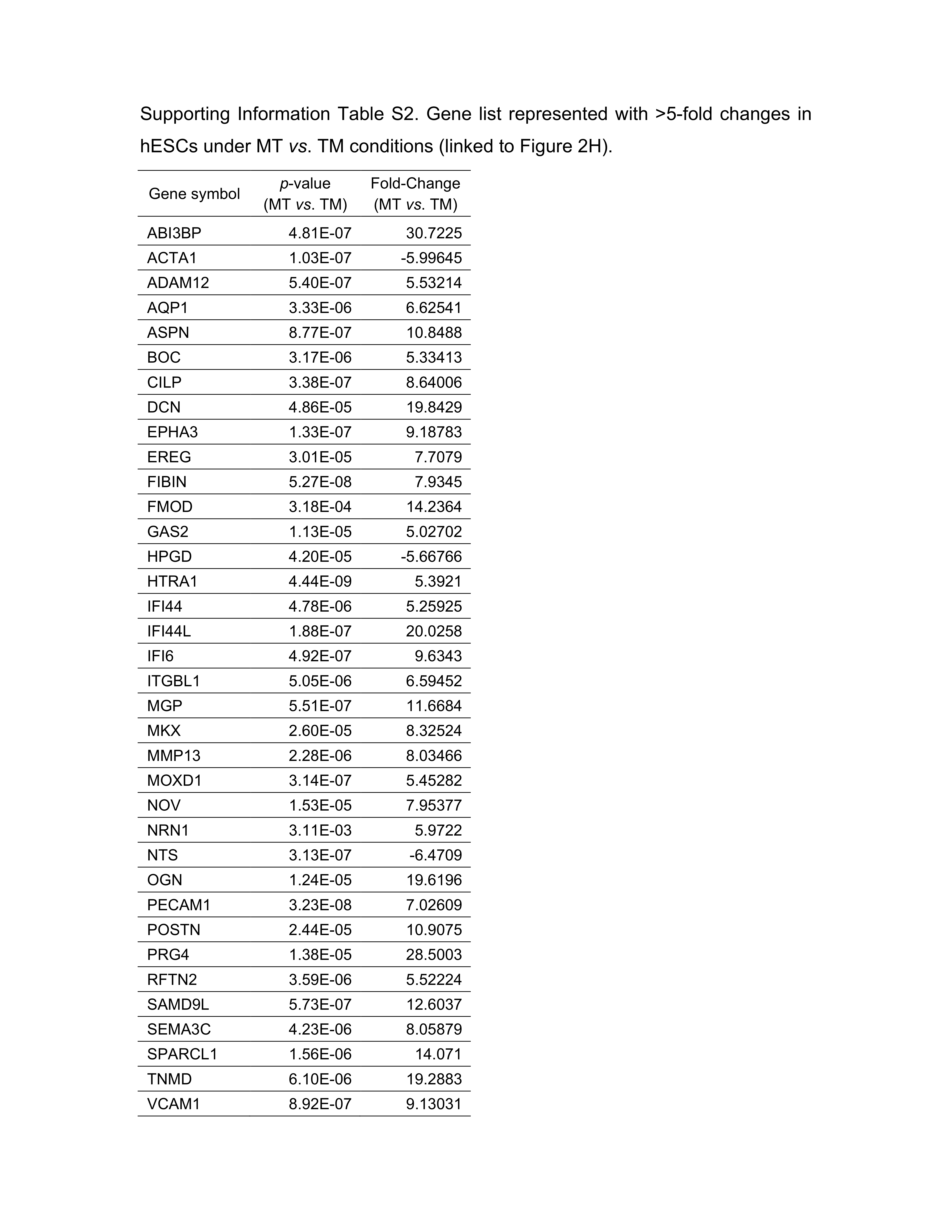

Supplement: Supplementary file 8 — Supporting Table 2 [file stem0033-1142-sd8.tiff]
