# Supplementary material for: Reversible Lineage-Specific Priming of Human Embryonic Stem Cells Can Be Exploited to Optimize the Yield of Differentiated Cells
Source: Stem Cells. 2015 Jan 13;33(4):1142–52. doi: 10.1002/stem.1952 (PMC4413029; doi:10.1002/stem.1952)
Supplement: Supplementary file 7 — Supporting Table 1 [file stem0033-1142-sd7.pdf]

Supporting Information Table S1. Gene list represented with >5-fold changes in hESCs under MM *vs*. TT conditions (linked to Figure 1J).

Gene symbol	*p*-value (TT *vs*. MM)	Fold-Change (TT *vs*. MM)
A2M	1.53E-04	7.6556
ABI3BP	4.52E-07	31.8512
ADAM12	2.42E-08	17.8293
AEBP1	3.75E-09	6.48097
ANO1	2.35E-07	10.1951
AQP1	5.10E-07	13.4175
ASPN	6.31E-07	12.4275
BGN	3.65E-04	7.82325
BICC1	1.97E-05	6.35259
C1QTNF3	3.68E-05	87.5644
C6orf204	1.97E-08	-5.47129
C9orf150	5.04E-07	8.53114
CCDC80	2.98E-06	6.0822
CHRNA1	3.90E-04	12.5897
CILP	3.74E-08	22.6456
COL11A1	4.71E-08	7.20128
COL12A1	2.25E-07	12.5794
CSRP1	8.32E-07	8.17857
CYBRD1	5.37E-08	10.0507
CYTL1	6.72E-08	17.9079
DAB2	1.41E-04	5.36682
DCN	8.27E-06	57.8495
DDR2	6.46E-06	10.5446
DDR2	8.32E-06	5.22352
EMP1	5.87E-06	13.6769
EPHA3	5.16E-08	13.4449
F2RL2	1.40E-03	12.7155
FBLN5	9.79E-07	16.9947
FBN1	4.29E-06	13.7573
FBN2	2.91E-07	5.99413
FGF14	3.75E-07	8.9736
FIBIN	4.38E-08	8.46539
FMOD	4.34E-06	11.5495
FNDC1	3.80E-06	10.1241
FSTL5	2.20E-05	9.34177
GAS2	2.35E-06	8.24004
GCG	6.13E-09	51.6015

GFRA1	1.75E-05	5.08965
GREM1	6.05E-06	5.47355
HIST1H2BK	9.09E-05	7.44737
HTRA1	1.24E-09	8.0341
IFI44L	5.48E-07	12.2235
IFI6	9.85E-07	7.50246
INPP4B	7.64E-07	5.05621
ITGA11	1.86E-03	5.23797
ITGBL1	3.11E-06	7.75925
KCNIP1	5.87E-06	6.64099
KCNJ15	1.23E-04	7.90429
KIAA1462	3.78E-07	5.62331
LCP1	4.92E-04	-5.50281
LHFP	2.13E-05	9.92178
LHX9	5.10E-06	11.8781
LOX	1.64E-04	8.77894
LPAR1	5.00E-07	6.59494
LRRC17	4.52E-07	26.6022
LTBP1	1.98E-09	5.45608
LTBP2	2.55E-06	5.85706
MAP2	1.64E-06	5.08322
MEOX2	9.89E-06	18.0503
MFAP4	7.88E-07	12.4143
MFAP5	8.29E-06	8.47154
MGP	4.73E-07	12.4394
MIR199A2	2.14E-05	9.93579
MKX	8.95E-06	12.7648
MMP13	4.21E-06	6.5349
MMP16	2.68E-05	5.23776
MSC	7.41E-07	8.41691
MXRA5	3.55E-06	9.09931
NCAM1	1.26E-07	9.75249
NNMT	1.37E-06	9.00686
NOV	1.43E-05	8.14667
NTS	3.67E-07	-6.15914
OGN	4.82E-06	33.0134
PECAM1	3.20E-09	17.6285
POSTN	6.58E-07	83.1552
PRG4	2.51E-06	88.5505
PRRX1	1.23E-06	13.8767
PRSS35	9.36E-06	5.9945

PTGS2	4.30E-06	7.38501
PTHLH	9.82E-09	8.64931
RANBP3L	3.01E-05	5.68855
RBM46	1.07E-04	6.71953
RFTN2	2.82E-06	5.9316
RHOJ	4.31E-05	13.9881
RNASE1	7.39E-06	-5.03162
SAMD9L	7.87E-07	11.0469
SCRG1	3.59E-07	15.0392
SEMA3C	1.68E-07	36.5573
SERPINB2	7.64E-05	33.8326
SERPINB7	5.31E-05	5.69817
SIX1	2.01E-05	9.60946
SLFN11	1.50E-06	8.86567
SRPX2	3.51E-05	11.0134
TBX18	2.73E-05	7.82912
THBS1	3.36E-05	6.37134
THBS2	1.85E-07	5.40931
THBS4	6.14E-04	9.87703
TIMP3	8.04E-04	5.36763
TM4SF1	3.03E-04	7.39077
TMPRSS11E	2.87E-07	-19.1907
TMPRSS11E	2.64E-07	-19.5547
TNFAIP6	6.51E-07	20.2009
TNMD	1.77E-06	38.5754
TRIL	4.69E-07	6.06212
TSPYL5	6.85E-08	6.72489
ZEB1	2.94E-06	6.12176
ZIC1	1.74E-07	9.62777
